# Cardiac arrest in a mother and daughter and the identification of a novel *RYR2* variant, predisposing to low penetrant catecholaminergic polymorphic ventricular tachycardia in a four‐generation Canadian family

**DOI:** 10.1002/mgg3.1151

**Published:** 2020-01-28

**Authors:** Matthew Tung, Filip Van Petegem, Samantha Lauson, Ashley Collier, Kathy Hodgkinson, Bridget Fernandez, Sean Connors, Rick Leather, Shubhayan Sanatani, Laura Arbour

**Affiliations:** ^1^ Royal Jubilee Hospital Victoria BC Canada; ^2^ Department of Biochemistry and Molecular Biology University of British Columbia Vancouver BC Canada; ^3^ Division of Medical Genetics Island Health Victoria BC Canada; ^4^ Provincial Medical Genetics Program Eastern Health St. John's NL Canada; ^5^ Clinical Epidemiology and Genetics, Faculty of Medicine Memorial University of Newfoundland St John's NL Canada; ^6^ Discipline of Genetics, Faculty of Medicine Memorial University of Newfoundland St John’s NL Canada; ^7^ Division of Cardiology Faculty of Medicine Memorial University of Newfoundland St John's NL Canada; ^8^ Division of Cardiology Department of Pediatrics University of British Columbia Vancouver BC Canada; ^9^ Department of Medical Genetics University of British Columbia Vancouver BC Canada; ^10^ Division of Medical Sciences University of Victoria Victoria BC Canada

**Keywords:** catecholaminergic polymorphic ventricular tachycardia, crystallography, *RYR2*, variable expression

## Abstract

**Background:**

Catecholaminergic polymorphic ventricular tachycardia (CPVT) is a rare inherited arrhythmia syndrome characterized by adrenergically driven ventricular arrhythmia predominantly caused by pathogenic variants in the cardiac ryanodine receptor (RyR2). We describe a novel variant associated with cardiac arrest in a mother and daughter.

**Methods:**

Initial sequencing of the *RYR2* gene identified a novel variant (c.527G > T, p.R176L) in the index case (the mother), and her daughter. Structural analysis demonstrated the variant was located within the N‐terminal domain of RyR2, likely leading to a gain‐of‐function effect facilitating enhanced calcium ion release. Four generation cascade genetic and clinical screening was carried out.

**Results:**

Thirty‐eight p.R176L variant carriers were identified of 94 family members with genetic testing, and 108 family members had clinical evaluations. Twelve carriers were symptomatic with previous syncope and 2 additional survivors of cardiac arrest were identified. Thirty‐two had clinical features suggestive of CPVT. Of 52 noncarriers, 11 had experienced previous syncope with none exhibiting any clinical features of CPVT. A documented arrhythmic event rate of 2.89/1000 person‐years across all carriers was calculated.

**Conclusion:**

The substantial variability in phenotype and the lower than previously reported penetrance is illustrative of the importance of exploring family variants beyond first‐degree relatives.

## Graphical abstract

Here, we present a family study and structural analysis of a likely pathogenic *RYR2* variant causing CPVT in a four‐generation Canadian family. If considering only the first‐degree relatives of our index case, the phenotype would seem to be highly penetrant and considered “severe.” However, with expanded, four‐generation genetic testing and clinical assessments (including 38 carriers of the variant) we show that the overall penetrance of the variant is low, illustrating the importance of exploring family variants beyond first‐degree relatives.

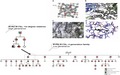

## 1 INTRODUCTION

Catecholaminergic polymorphic ventricular tachycardia (CPVT) is a rare inherited arrhythmia syndrome with an estimated population prevalence of 1:10,000 (Priori, Wilde, & Horie, 2013; Ackerman, Priori, & Willems, 2011). It is characterized by adrenergically driven ventricular arrhythmias, induced by physical or emotional stress in the absence of structural heart disease or a prolonged QTc interval (Leenhardt, Denjoy, & Guicheney, 2012; Leenhardt et al., 1995; Priori, Napolitano, & Memmi, 2002; Priori et al., 2013; Swan, Piippo, & Viitasalo, 1999). Resting ECGs are generally normal with the development of ventricular ectopy with exercise that can progressively increase in complexity to bidirectional ventricular tachycardia, polymorphic ventricular tachycardia, or ventricular fibrillation (Leenhardt et al., 2012, 1995; Priori et al., 2002; Swan et al., 1999).

Age of symptom onset is variable with presentations ranging from syncope to sudden death. Mortality is reported to be high with up to 30% of untreated individuals experiencing sudden cardiac death by the age of 30 (Coumel, 1978; Leenhardt et al., 1995; Priori et al., 2002; Swan et al., 1999). Pharmacological therapy with beta blockade is indicated for all diagnosed individuals resulting in cardiac event rates reduced to as low as 3% per year (Hayashi, Denjoy, & Extramiana, 2009). The addition of flecainide may also be used to reduce the burden of ventricular arrhythmia (Padfield, AlAhmari, & Lieve, 2016; Priori et al., 2013; Rosso, Kalman, & Rogowski, 2007; Werf, Kannankeril, & Sacher, 2011). Activity restriction is recommended along with implantation of an implantable cardiac defibrillator (ICD) for those who do not respond to optimal medical therapy (Priori et al., 2013).

Genetic testing is currently able to identify a disease‐causing variant in about 65% of patients with CPVT (Ackerman et al., 2011). Two autosomal dominant forms, due to pathogenic variants in the genes encoding for the cardiac ryanodine receptor, *RYR2* (CPVT1, MIM# 604772), and calmodulin, *CALM1* (CPVT4, MIM# 614916), and two autosomal recessive forms resulting from pathogenic variants in the genes encoding for cardiac calsequestrin, *CASQ2* (CPVT2, MIM# 611938) and triadin, *TRDN* (CPVT5, MIM# 615441) have been delineated (Eldar, Pras, & Lahat, 2002; Laitinen, Brown, & Piippo, 2001; Nyegaard, Overgaard, & Søndergaard, 2012; Priori, Napolitano, & Tiso, 2001). All four genes are involved in the tightly controlled cycling of intracellular calcium that provides the basis for regulation of cardiac contraction and also for arrhythmogenesis in CPVT via calcium overload and delayed after depolarizations (Liu, Colombi, & Memmi, 2006).

Pathogenic variants in *RYR2* are the most frequently identified, accounting for 50%–55% of all pathogenic variants in CPVT (Priori et al., 2002). Cardiac ryanodine receptors (RyRs) are responsible for calcium release from the sarcoplasmic reticulum in response to calcium entry from the dihydropyridine receptor with pathogenic variants in highly conserved regions identified and co‐segregating in typical CPVT phenotypes (Fabiato, 1983; Priori et al., 2002, 2001). Multiple pathogenic variants have been shown to facilitate store overload‐induced calcium release (Chen, Wang, & Chen, 2014; Jiang, Xiao, & Yang, 2004). RyR2 has been studied extensively via structural methods (Pancaroglu & Van Petegem, 2018). Cryo‐EM structures of full‐length pig RyR2 have been reported with a resolution up to 3.6Å (Peng et al., 2016; GongJuly, Chi, & Wei, 2019). Several areas in the cytosolic shell suffer from low local resolution, but high‐resolution crystal structures are available for several RyR2 cytosolic domains, including the N‐terminal region (Kimlicka, Tung, et al., 2013), phosphorylation domain (Haji‐Ghassemi, Yuchi, & Van Petegem, 2019; Yuchi, Lau, & Van Petegem, 2012), and two SPRY domains (Lau & Van Petegem, 2014; Yuchi, Yuen, & Lau, 2015). Mapping disease‐associated variants show that most are located close to the central four‐fold symmetry axis, extending from the cytosolic cap to the transmembrane region, with only few variants located further away (Pancaroglu & Van Petegem, 2018). The N‐terminal region forms a gating ring (Kimlicka, Lau, Tung, & Van Petegem, 2013), and pathogenic variants in this area have been suggested to destabilize intersubunit interactions either directly or indirectly by affecting a central anion‐binding site (Kimlicka, Tung, et al., 2013).

Exercise stress testing (EST), although the primary clinical screening tool, has limited sensitivity in detecting CPVT. Arrhythmia during exercise is the clinical hallmark of CPVT, but genotype/phenotype correlational studies have demonstrated only 50%–60% sensitivity for predicting carrier status and also an inability to comprehensively risk stratify for cardiac events in genotype‐positive patients (Bauce, Rampazzo, & Basso, 2002; Hayashi et al., 2009; Hayashi, Denjoy, & Hayashi, 2012; Priori et al., 2002). Exploration of multigenerational families provides a valuable opportunity to understand the lifelong spectrum of clinical features associated with a single disease‐causing variant. Here, we present a four‐generation family with a novel *RYR2* variant (c.527G > T, p.R176L) identified after cardiac arrests in a mother and daughter.

## 2 MATERIALS AND METHODS

### 2.1 Case description

The index case, a 43‐year‐old female came to attention while her daughter was being evaluated after a cardiac arrest while on an amusement park ride. The index case had a history of two previous cardiac arrests: at age 28 during surgery and at age 32 during physical exertion and emotional stress. A finding of transiently reduced ejection fraction on echocardiography following the second cardiac arrest raised the possibility of an underlying cardiomyopathy or myocarditis. Normalized left ventricular systolic function, however, was documented 3 months later. Investigations for arrhythmogenic right ventricular cardiomyopathy (ARVC) after the first cardiac arrest ruled out the common founder haplotype for the gene most often causing ARVC in her home region of Newfoundland (*TMEM43* p.S358L) (Merner, Hodgkinson, & Haywood, 2008).

Early investigations on her daughter focused on the previous consideration of the presumed familial diagnosis of ARVC, and long QT syndrome (LQTS) because of a postcardiac arrest prolonged QTc interval. The five gene ARVC panel (*DSC2, DSG2, DSP, PKP2, TMEM43*) carried out on the daughter was negative; however, she was found to carry a previously thought to be disease‐causing *KCNQ1* variant (p.R518Q) on the 10 gene LQTS panel (*KCNQ1, KCNH2, SCN5A, ANK2, KCNE1, KCNE2, KCNJ2, CACNA1C, CAV3, SCN4B*). Therefore, LQTS1 was initially considered the most likely cause of cardiac arrest in the daughter. Subsequent testing on the mother surprisingly showed she did not carry the *KCNQ1* variant, leaving her recurrent cardiac arrests unexplained. Her asymptomatic son also harbored the *KCNQ1* variant, suggesting it was inherited from the father. The p.R518Q variant has been since re‐evaluated and is currently considered a variant of uncertain significance (Variation ID 67036, https://www.ncbi.nlm.nih.gov/clinvar/variation/67036/).

**Figure 1:**
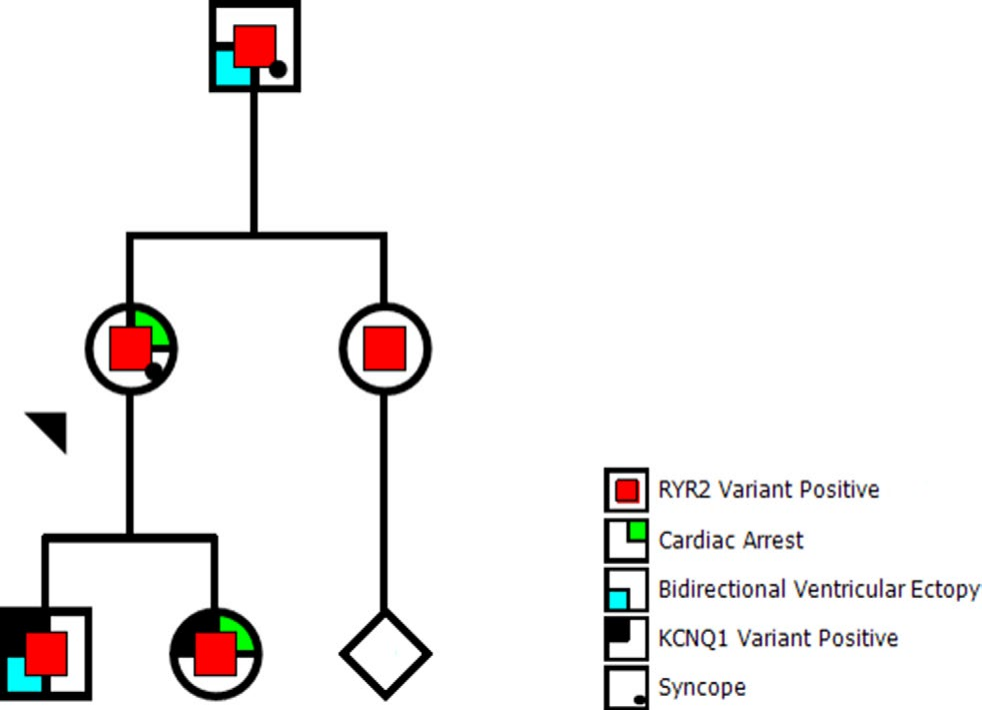
Pedigree of first‐degree relatives of the index case. Index case indicated by black triangle. Red central symbols indicate positive status for the *RYR2* variant. Green upper right quadrant indicates a previous cardiac arrest. Blue lower left quadrant designates bidirectional ventricular ectopy. Black upper left quadrant represents positive status for the *KCNQ1* variant. Lower left quadrant dot indicates syncope. Diamond represents additional sibling(s)

**Figure 2:**
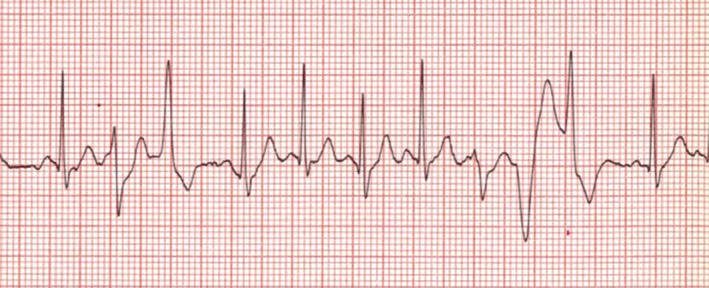
Post‐genetic testing exercise stress test demonstrating bidirectional premature ventricular ectopy with exercise, consistent with a CPVT phenotype in the son of the index case

Subsequent investigations of the index case to complete an ARVC panel included sequencing of the *RYR2* gene, which then identified a novel variant (NM_001035.2:c.527G > T, p.R176L) of unknown clinical significance, but assessed to be “likely pathogenic” with in silico programs (Variation ID 201195, https://preview.ncbi.nlm.nih.gov/clinvar/variation/201195). The son and daughter were then tested and both were found to be carriers of this *RYR2* variant (Figure 1). Only the son was capable of completing a postgenetic testing EST, which demonstrated bidirectional premature ventricular ectopy with exercise, consistent with a CPVT phenotype (Figure 2). Of note, three preceding ESTs demonstrated only isolated premature ventricular contractions (PVCs).

### 2.2 Study population and clinical screening

After identification of the novel variant in the index case and her two children, a four‐generation pedigree was completed and cascade genetic testing was carried out for both the *KCNQ1* and the *RYR2* variants. Clinical evaluation was performed in 108 family members (four generations). Medical records were reviewed specifically for resting 12 lead ECG, transthoracic echocardiography (TTE), and EST. QT intervals from 12 lead ECGs were corrected for rate using Bazett's formula. EST results were analyzed and scored using a ventricular arrhythmia score and reviewed for the presence of PVCs, bidirectional ventricular beats and ventricular tachycardia (Werf et al., 2011).

Chart reviews were performed to establish cardiac symptom status, age of symptom onset, ICD, and use of CPVT‐directed pharmacological therapy. Consent for the index family study was obtained by the index case, with research ethics board waiver received through Island Health. The extended family members in Newfoundland provided individual consent for genotyping and medical record review (Memorial University of Newfoundland HREB study no. 00.176).

### 2.3 Structural Analysis

The position of the R176L variant was located on the crystal structure of the RyR2 N‐terminal disease hot spot (Kimlicka, Tung, et al., 2013) (PDB ID 4L4H) and on the 3.6Å resolution RyR2 cryo‐EM structure (Gong et al., July, 2019) (PDB ID 6JI8). All images were generated using Pymol (Schrodinger).

## 3 RESULTS

**Figure 3:**
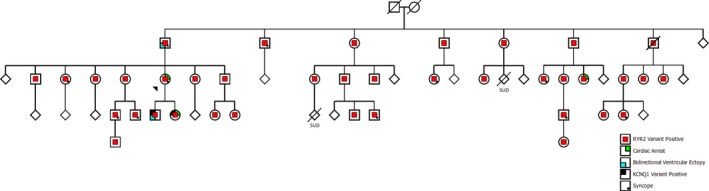
Expanded pedigree of the family of the index case. Index case indicated by black triangle. Red central symbols indicate positive status for the *RYR2* variant. Green upper right quadrant indicates a previous cardiac arrest. Blue lower left quadrant designates bidirectional ventricular ectopy. Black upper left quadrant represents positive status for the *KCNQ1* variant. Lower left quadrant dot indicates syncope. Diamond represents additional sibling(s)

In all, 108 members of the family were clinically screened with 94 members undergoing genetic testing. Thirty‐eight were confirmed to be carriers of the p.R176L variant. Sixty‐two members of the family (including 18 p.R176L carriers) were also screened for the *KCNQ1* (p.R518Q) variant detected in the index case's two children. No additional carriers of the *KCNQ1* variant were identified. Baseline characteristics for the 38 p.R176L variant carriers are shown in Tables S1 and S2, and expanded pedigree (Figure 3).

Analysis of the family pedigree identified two relatives of the index case who experienced sudden cardiac arrest (SCA) and two relatives who suffered sudden unexplained death (SUD). The two cases of SCA consisted of her daughter and a paternal cousin (both carriers of p.R176L). The two cases of SUD occurred in another paternal cousin (carrier status unknown at time of death—25% risk) and their son (carrier status unknown at time of death—12.5% risk). Other sudden cardiac deaths 12.5% risk or less from a carrier were documented but not included in this analysis.

Of the 38 documented p.R176L carriers, 12 were symptomatic with previous syncopal episodes, and 3 of those were assessed to have had seizures. The presentation of the condition, associated with the p.R176L variant, was consistent with an autosomal dominant pattern with reduced penetrance and variable expressivity through the index case's paternal kindred.

All 38 carriers underwent TTE, and three also had cardiac MRI. One carrier had mild right ventricular dilation noted with no evidence of any other structural heart disease on the remaining TTEs or MRIs.

Thirty‐two of the 38 carriers underwent EST with nine having a ventricular arrhythmia score of ≥2 including 4 of the 12 carriers who had experienced a syncopal episode. An additional eight had isolated PVCs during EST.

**Figure 4:**
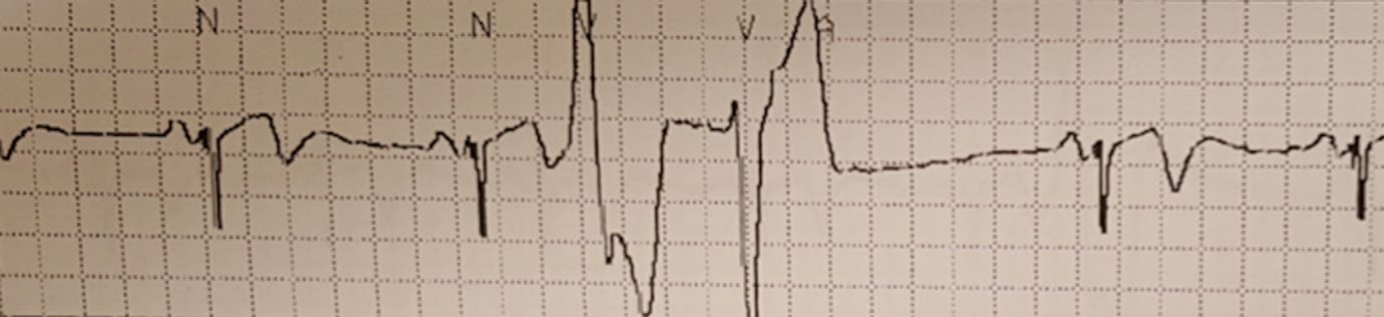
Electrocardiogram of the index case's father, carrier of the p.R176L variant, demonstrating bidirectional premature ventricular contractions. This tracing was recorded during his admission for an acute coronary syndrome 7 years prior to the index case presenting with cardiac arrest

ECG review was negative for evidence of LQTS in all 38 carriers including the son and daughter of the index case (with the exception of her immediate postcardiac arrest ECGs). The 76–year‐old father of the index case (a carrier of p.R176L), upon retrospective chart review, had subtle clinical features of CPVT present several years prior to the identification of the variant. In particular, bidirectional PVCs were present during admission with an acute coronary syndrome in 1995 (Figure 4).

Co‐Morbidity: Two variant carriers had a history of coronary artery disease (CAD) and were treated with beta‐blockers for this indication.

Noncarriers of the variant: 46 relatives did not carry the p.R176L variant based on genetic testing. An additional 24 relatives did not undergo genetic testing due to varying reasons including being obligate noncarriers (6), being lost to follow up, or declining genetic testing.

Of 52 confirmed not to be carriers of the p.R176L (46 tested, 6 obligate noncarriers) none had experienced a cardiac arrest, 11 had previous syncope, 27 had undergone EST with 3 demonstrating isolated PVCs only.

Diagnostic test results and patient characteristics for variant carriers are included in Tables S1 and S2.

## 4 STRUCTURAL ANALYSIS

**Figure 5:**
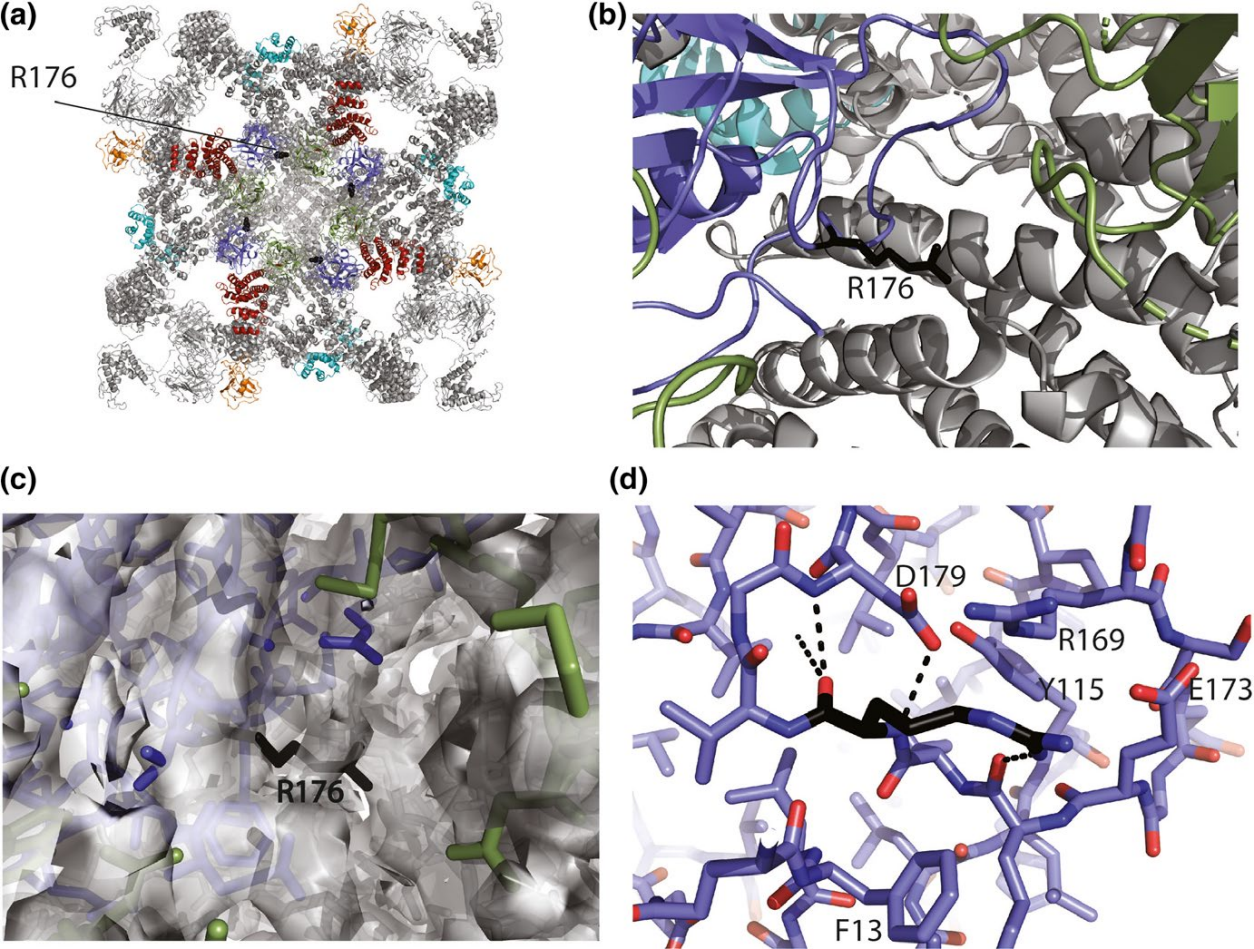
Structural mapping of the R176 residue. A. Cryo‐EM structure of RyR2 (PDB ID 6JI8) showing the overall position of R176 in black. Domains in the N‐terminal disease hot spot are colored: blue, Domain A; green, Domain B; red, Domain C. Two auxiliary proteins are also shown in color; FKBP12.6 (orange) and Calmodulin (cyan). B. Close‐up of the R176 residue, showing that it is in the vicinity of two interfaces, including an interface with domain B of a neighboring subunit (green), and also with a C‐terminal area (gray helices). C. Cryo‐EM density map of the structure shown in panels A and B shows that the local resolution around R176 is low, and its precise chemical environment cannot be interpreted directly. D. Crystal structure of the N‐terminal disease hot spot of RyR2 (PDB ID 4L4H), showing the chemical environment of R176 (black). Several residues involved in packing around R176 are labeled. Black dotted lines indicate hydrogen bonds

RyRs form large homotetrameric channels, consisting of six transmembrane helices per subunit and a large cytosolic assembly (Yuchi & Van Petegem, 2016). The p.R176L variant is located within the N‐terminal domain of RyR2, which is part of the first disease hot spot*.* This hot spot forms a cytosolic vestibule, forming a ring around the four‐fold symmetry axis, directly at the cytosolic surface (Figure 5a,b). More specifically, the p.R176L resides in a loop that has been found to contain positions for multiple disease variants, thus termed the “hot spot loop” (Amador, Liu, & Ishiyama, 2009; Lobo & Van, 2009). The local resolution around R176 in the cryo‐EM structure of RyR2 is too low to see the precise interactions it makes with neighboring residues (Figure 5c). However, crystal structures of the N‐terminal domains show that the wild‐type residue, R176, is involved in an extensive network that dictates the conformation of the loop (Figure 5d). All of these would be lost with the p.R176L substitution, resulting in substantial conformational changes within the loop (Amador, Kimlicka, & Stathopulos, 2013). Indeed, the more subtle variant p.R176Q, which still allows for hydrogen bond capabilities, was already shown to result in conformational changes of the loop (Amador et al., 2013). Changing the loop conformation is expected to directly affect the ability of the channel to open. Previously, it has been suggested that the loop containing p.R176Q forms interactions with another subunit (Kimlicka, Lau, et al., 2013). During opening and closing of the channel, these interactions are disrupted, thus resulting in an energetic barrier for channel opening (Kimlicka, Lau, et al., 2013; Zhong, Liu, & Zhu, 2013). Changing the conformation of the loop is thus expected to weaken intersubunit interactions, thus reducing the barrier and resulting in facilitated opening. Indeed, several disease‐causing variants near this intersubunit interface have been shown to be gain‐of‐function (Kimlicka, Lau, et al., 2013). In addition, the R176 residue is close to an interface with the C‐terminal area (Figure 5b), and the altered conformation of the loop may thus also stabilize this interaction. As such, the structural analysis suggests p.R176L is likely a gain‐of‐function variant, leading to facilitated channel opening and premature or prolonged release of calcium ions.

## 5 DISCUSSION

There are more than 150 unique, mostly missense, pathogenic variants identified in the *RYR2* gene as the underlying cause for CPVT (Ackerman et al., 2011; Leenhardt et al., 2012; Priori et al., 2001; Stenson, Ball, & Mort, 2003; Stenson et al., 2014). Our clinical and laboratory assessment supports the pathogenicity of the p.R176L variant as another cause for CPVT associated with sudden cardiac arrest. Based on the majority of cohorts and extended families described, pathogenic variants causing CPVT are associated with high penetrance and variable expressivity. More recently, however, findings from a large multigenerational Spanish family study demonstrated that pathogenic variants can be associated with lower penetrance than previously reported (Wanguemert, Bosch Calero, & Perez, 2015). Our study supports this phenomenon. Here, we review clinical information on 38 variant carriers and their family members in four generations demonstrating substantial variability in phenotype associated with the variant and considerably lower penetrance consistent with this recent report. Notably, if considering only the first‐degree relatives of our index case, the phenotype would seem to be highly penetrant and malignant (Figure 1). When considering all variant carriers, the terms “predisposition” or “susceptibility,” would seem to be more appropriate.

In contrast, a multicenter cohort of 140 patients in the Netherlands (24 index cases, 116 relatives positive for the variant), 54 (38%) of the relatives had a clinical diagnosis of CPVT with a further eleven having isolated PVCs not meeting the definition of the clinical diagnosis (Werf, Nederend, & Hofman, 2012). Collectively, the arrhythmic event rate was 21.7/1000 person‐years in the index cases and 4.4/1000 person‐years among relatives with *RYR2* variant.

Variable expression in CPVT has been illustrated in a description of an extended family with CPVT due to a p.G14876A variant in *RYR2.* In this family with an apparent highly penetrant variant, 10 of 11 variant carriers experienced syncope (including two sudden cardiac deaths during sleep) and were also found to have arrhythmia during EST ranging from isolated PVCs to ventricular fibrillation (Allouis, Probst, Jaafar, Schott, & Le Marec, 2005).

We report in our extended family an arrhythmic event rate of 2.89/1000 person‐years in variant carriers (index and relatives combined). One hundred and eight members of the family were clinically screened with 94 undergoing genetic testing with 38 variant carriers identified. Three variant carriers had experienced sudden cardiac arrest, twelve had experienced syncope and nine had evidence of CPVT during EST (ventricular arrhythmia score ≥2). To date, of the 32 variant carriers that completed an EST, 11 have not experienced syncope or had evidence of even minor arrhythmia during exercise (ventricular arrhythmia score ≤1).

The RyR2 channel plays an important role in cardiac excitation–contraction coupling. During the plateau phase of the cardiac action potential, calcium influx in the sarcolemma triggers calcium to be released from the sarcoplasmic reticulum through the RyR2 channel. This released calcium binds to troponin C and results in myocardial contraction. Based on its location in the hot spot loop of the N‐terminal domain of RyR2, the p.R176L variant in *RYR2* likely results in increased spontaneous diastolic calcium release which is predicted to produce delayed after depolarizations leading to arrhythmogenesis associated with increased heart rates and sympathetic tone—a hallmark of CPVT.

The son and daughter of the index case were the only members of the family found to carry both the *RYR2* and *KCNQ1* variants and along with their mother had the clearest CPVT phenotypes (all three underwent ICD implantation in conjunction with beta blockade). The concept of clinical phenotype heterogeneity resulting from genetic modifiers has been described in LQTS, Brugada syndrome, hypertrophic cardiomyopathy and ARVC, with modifiers ranging from common single nucleotide polymorphisms to compound pathogenic variants (Baroudi, Acharfi, Larouche, & Chahine, 2002; Crotti, Lundquist, & Insolia, 2005; Crotti, Monti, & Insolia, 2009; Itoh, Shimizu, & Hayashi, 2010; Kapplinger et al.,; Nof, Cordeiro, & Pérez, 2010; Villiers, Merwe, & Crotti, 2014; Viswanathan, Benson, & Balser, 2003; Xu, Yang, & Vatta, 2010). Given *KCNQ1* variants are associated with long QT syndrome 1 (LQTS1), which is also characterized by exertional ventricular arrhythmia, it would seem plausible for this variant to potentially exert a modifying effect on phenotype expression (Schwartz, Crotti, & Insolia, 2012).

The variability seen in phenotype expression is also a product of the limitations of our clinical screening tests with the limited sensitivity of EST reflected by the three ESTs that were negative in the index case's son before a positive result was eventually seen on the fourth EST.

In conditions such as CPVT where even the best predictive tests, both clinical and genetic, have limited prognostic value, cross sectional family studies add to our ability to counsel within families about the range of potential outcomes particularly when variant carriers span multiple generations, with varying degrees of separation from the index case and a broad range of ages. For instance, in our family, the 11 members with no clinical or EST features of CPVT span the ages of 10–55 and range from 1 to 3 degrees of separation from the index case. However, if we had only considered the nuclear family presenting, the associated risk with the variant would have been considered quite high. Multigenerational genotyping and longitudinal follow‐up of such families offers the opportunity to further characterize variable expression and nonpenetrance associated with “silent” variant carriers and aid refinement of genotype/phenotype correlations derived from larger more generalized cohorts of CPVT patients.

The low arrhythmic event rate among variant carriers seen in our large multigenerational family raises questions about how variant carriers should be counseled, labeled, and managed. With increasing access to genetic testing, the gathering of individual genomic data will continue to accelerate and likely outstrip the pace of progress of our understanding of the prognostic implications of many genetic testing results. Treatment of concealed patients positive for the familial variant with beta‐blockers is suggested by guidelines as a measure “that can be useful” with recommendations based on studies observing higher arrhythmic event rates off beta‐blockers in both probands and relatives carrying the familial variant (Hayashi et al., 2009; Priori et al., 2013; Werf et al., 2012). These studies, however, had much higher event rates than our familial cohort with a 27% of 8‐year event rate reported by Hayashi et al in the cohort using beta‐blockers and a 58% event rate in those not using beta‐blockers (Hayashi et al., 2009).

Given the potentially devastating consequences of an arrhythmic event and the relative safety of beta‐blockers we agree with the guidelines but would propose an approach emphasizing to the patients the concept of genetic susceptibility rather than a “diagnosis” of CPVT when a variant is present but features of disease are absent. We propose framing discussions about pharmacological therapy in terms of prophylaxis and monitoring rather than “treatment,” with counseling underpinned and informed by knowledge of the family's variant‐specific event rate when possible.

In conclusion, our clinical and laboratory study of a four‐generation family with a novel *RYR2* (c.527G > T, p.R176L) variant provided a unique opportunity to explore the mechanisms of disease, penetrance, and variable presentation in this rare, but highly malignant inherited arrhythmia condition.

The low arrhythmic event rate in our variant positive relatives highlights the variable expression and penetrance of CPVT and the need for a personalized approach to genetic counseling and medical management.

## CONFLICT OF INTERESTS

MT, FVP, SL, AC, KH, BF, SC, RL, SS, LA, declare no conflict of interest.

## Data Availability

The *RYR2* variant presented in this study has been submitted to ClinVar, and is available at https://preview.ncbi.nlm.nih.gov/clinvar/variation/201195 (Variation ID 201195).
