# Supplementary material for: Cardiac arrest in a mother and daughter and the identification of a novel RYR2 variant, predisposing to low penetrant catecholaminergic polymorphic ventricular tachycardia in a four‐generation Canadian family
Source: Mol Genet Genomic Med. 2020 Jan 28;8(4):e1151. doi: 10.1002/mgg3.1151 (PMC7196448; doi:10.1002/mgg3.1151)
Supplement: Supplementary file 1 [file MGG3-8-e1151-s001.docx]

**Supporting Information**

**Table 1.** Baseline characteristics of British Columbia branch *RYR2* mutation carriers including the index case.

| Patient# | Age | Sex | Carrier of *RYR2* R176L | Carrier of *KCNQ1* R518Q | Proband/ relative | Syncope | Aborted Cardiac Arrest | Sudden cardiac death | Symptoms on exertion/stress | Age at first symptom | EST Ventricular arrhythmia score † | QTC (date) | Betablocker therapy | ICD |
| --- | --- | --- | --- | --- | --- | --- | --- | --- | --- | --- | --- | --- | --- | --- |
| 1 | 44 | F | + | - | Proband | + | + | _ | + | 14 | 2 | 360ms (Dec 2014)  398ms (Oct 2008)  422ms (Feb 1991) | + | + |
| 2 | 22 | F | + | + | Relative | - | + | - | + | 16 | 1 | 450ms (Oct 2009)  442ms (Feb 2011) | + | + |
| 3 | 16 | M | + | + | Relative | - | - | - | NA | NA | 4 | 384ms (Jan 2014)  400ms (Jan 2012)  390ms (Jul 2011) | + | + |
| 4 | 45 | F | + | - | Relative | - | - | - | NA | NA | 3 | 401ms (Sept 2013)  388ms (May 2010)  396ms (Jan 2011) | + | - |
| 5 | 26 | M | + | NA | Relative | + | - | - | + | 15 | 1 | 416ms (July 2013)  370ms (May 2010) | - | - |
| 6 | 5 | M | + | NA | Relative | - | - | - | NA | NA | NA | NA | - | - |
| 7 | 24 | M | + | NA | Relative | + | - | - | + | 14 | 1 | 412ms (Nov 2013)  404ms (May 2010) | - | - |

†1, absent or isolated VPBs (ventricular premature beats); 2, bigeminal VPBs and/or frequent VPBs [>10 per min]; 3, couplet; and 4, non-sustained ventricular tachycardia [NSVT], >3 successive VPBs)

**Table 2**. Characteristics of Newfoundland branch of *RYR2* variant carriers.

| Patient # | Age | Sex | Carrier *RYR2* R176L | Carrier *KCNQ1* R518Q | Syncope | Aborted Cardiac Arrest | Sudden cardiac death | Symptoms on exertion/stress | Age at first symptom | Ventricular arrhythmia score with EST | QTC (date) | Betablocker therapy | ICD |
| --- | --- | --- | --- | --- | --- | --- | --- | --- | --- | --- | --- | --- | --- |
| 11072.101 | 76 | M | + | - | + | - | - | - | 61 | 2 | 388ms (Jan 2016)  374ms (Mar 2015)  385ms (Mar 2014)  420ms (Nov 1996) | + | - |
| 11072.001 | 49 | M | + | - | - | - | - | NA | NA | 1 | 383ms (Dec 2014)  388 (Oct 2010)  382 (Sep 2010) | - | - |
| 11072.003 | 44 | F | + | - | - | - | - | NA | NA | 2 | 389ms (Feb 2015)  395ms (Jan 2013) | - | - |
| 11072.004 | 38 | M | + | - | - | - | - | NA | NA | 1 | 377ms (Aug 2013) | - | - |
| 11072.006 | 51 | F | + | - | + | - | - | + | ~25 | 1 | 429ms (Apr 2014) | + | - |
| 11072.007 | 52 | F | + | - | - | - | - | NA | NA | 1 | 396ms (Dec 2015)  427ms (Feb 2014) | + | - |
| 11072.106 | 80 | M | + | - | - | - | - | NA | NA | NA | 405ms (May 2014)  425ms (Jan 2009) | + | - |
| 11072.A10 | 22 | F | + | NA | - | - | - | NA | NA | NA | 367ms (July 2015) | - | - |
| 11072.A22 | 13 | F | + | NA | - | - | - | NA | NA | 1 | 400ms (Jan 2015)  381ms (Nov 2013)  427ms (Dec 2010) | - | - |
| 11072.A23 | 13 | F | + | NA | - | - | - | NA | NA | 1 | 400ms (Nov 2013)  408ms (Nov 2012)  432ms (Dec 2010) | - | - |
| 11072.108 | 56 | F | + | - | + | - | - | - | ~25 | 1 | 391ms (June 2015)  403ms (Jan 2015)  399ms (Nov 2014) | + | - |
| 11072.029 | 45 | F | + | - | - | - | - | NA | NA | 1 | 406ms (Jan 2015) | + | - |
| 11072.A52 | 65 | M | + | NA | - | - | - | NA | NA | 1 | 386ms (Jan 2016)  376ms (Feb 2015) | - | - |
| 11072.053 | 70 | M | + | - | + | - | - | - | 5? | 2 | 394ms (Nov 2015)  390ms (May 2013)  418ms (Jan 2012) | - | - |
| 11072.057 | 48 | F | + | - | - | - | - | NA | NA | 1 (0) | 420ms (June 2015)  404ms (Nov 2014)  428ms (Mar 2014) | - | - |
| 11072.034 | 51 | F | + | - | - | + | - | - | 26 | 2 | 435ms (Aug 2015)  457ms (Aug 2015)  402ms (Feb 2013) | + | + |
| 11072.A34 | 34 | M | + | NA | + | - | - | - | ~5 | 1 | 384ms (Aug2014) | - | - |
| 11072.B34 | 8 | F | + | NA | - | - | - | NA | NA | NA | 394ms (Oct 2014) | - | - |
| 11072.104 | 67 | F | + | - | - | - | - | NA | NA | 3 | 377ms (Dec 2015)  409ms (Feb 2010)  388ms (Apr 2008) | + | - |
| 11072.010 | 55 | F | + | NA | - | - | - | NA | NA | 1 (0) | 372ms (Apr 2014) | + | - |
| 11072.A31 | 48 | M | + | NA | - | - | - | - | NA | 1 | 417ms (June 2015)  387ms (Mar 2015) | + | - |
| 11072.002 | 18 | F | + | NA | + | - | - | - | - | NA | 373ms (June 2015) | + | - |
| 11072.T24 | 44 | M | + | - | - | - | - | NA | NA | 1 (0) | 383ms (Apr 2014) | - | - |
| 11072.A27 | 16 | F | + | - | - | - | - | NA | NA | 1 (0) | 377ms (Sep 2015)  356ms (Oct 2015)  356ms (Jul 2014) | - | - |
| 11072.B52 | 38 | M | + | NA | + | - | - | ? | ? | 2 | 360ms (Mar 2015) | + | - |
| 11072.C52 | 15 | F | + | NA | - | - | - | NA | NA | 1 (0) | 388ms (Mar 2015) | + | - |
| 11072.C53 | 13 | F | + | NA | - | - | - | NA | NA | 1 (0) | 368ms (Mar 2015)  382ms (Feb 2015) | + | - |
| 11072.C54 | 10 | M | + | NA | - | - | - | NA | NA | 1 (0) | 390ms (Nov 2015)  385ms (Mar 2015) | + | - |
| 6250.102 | 43 | F | + | - | + | - | - | - | 15 | 1 (0) | 375ms (May 2015) | - | - |
| 6250 | 17 | M | + | - | - | - | - | NA | NA | 1 | 397ms (Jan 2016)  411ms (Aug 2015)  390ms (Jan 2015) | - | - |
| 11072.B01 | 25 | F | + | - | - | - | - | NA | NA | NA | 433ms (Nov 2015)  340ms (Dec 2014) | - | - |
